# Beyond Motor Neurons in Spinal Muscular Atrophy: A Focus on Neuromuscular Junction

**DOI:** 10.3390/ijms25137311

**Published:** 2024-07-03

**Authors:** Francesca Torri, Michelangelo Mancuso, Gabriele Siciliano, Giulia Ricci

**Affiliations:** Department of Clinical and Experimental Medicine, University of Pisa, 56126 Pisa, Italy; francesca.torri@phd.unipi.it (F.T.);

**Keywords:** SMA, neuromuscular junction, fatigue, therapy, pyridostigmine, salbutamol

## Abstract

5q-Spinal muscular atrophy (5q-SMA) is one of the most common neuromuscular diseases due to homozygous mutations in the *SMN1* gene. This leads to a loss of function of the *SMN1* gene, which in the end determines lower motor neuron degeneration. Since the generation of the first mouse models of SMA neuropathology, a complex degenerative involvement of the neuromuscular junction and peripheral axons of motor nerves, alongside lower motor neurons, has been described. The involvement of the neuromuscular junction in determining disease symptoms offers a possible parallel therapeutic target. This narrative review aims at providing an overview of the current knowledge about the pathogenesis and significance of neuromuscular junction dysfunction in SMA, circulating biomarkers, outcome measures and available or developing therapeutic approaches.

## 1. Introduction

**Figure 1:**
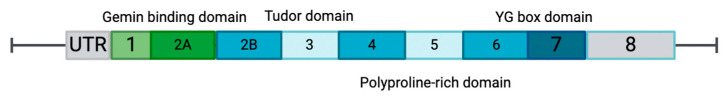
Schematic representation of SMN protein and its domains: the N-terminal Gemin binding domain is necessary for interaction with Gemin proteins, which are involved in snRNPs biogenesis; the Tudor domain, which is not essential for cells viability; a polyproline-rich domain; and the YG box domain, a self-interaction domain.

5q-Spinal muscular atrophy (5q-SMA) is one of the most common neuromuscular diseases [1] due to homozygous mutations in the *SMN1* gene—mostly deletions of exons 7–8 followed by the deletion of other exons and point mutations [1]. This leads to a loss of function of the *SMN1* gene, which in the end determines lower motor neuron degeneration [1]. The SMN protein is involved in the assembly of spliceosomal small nuclear ribonucleoproteins (snRNPs), which are in turn required for pre-mRNA maturation and splicing. Deletions of exons 7 and 8 affect the C-terminal domain of the protein, which is then more prone to degradation, impairs interactions with other proteins and has an altered capacity to regulate the splicing process of other pre-mRNAs. Thus, the overall pleotropic function of SMN leads to widespread abnormalities in neuronal biology [2,3,4,5]. The SMN protein’s structure is described in Figure 1. Broader effects of SMN loss in other tissues such as peripheral nerves and muscles have been described in terms of impaired myelination, axonal degeneration, and muscle damage secondary to denervation as well as primary biological impairment involving myogenesis and regeneration [6,7]. The SMN protein, in fact, is involved in the regulation of vital processes such as miRNAs biogenesis, RNA transcription and RNA translation, among others, and its complete absence is not compatible with embryonic development [8]. In the human species, an almost identical copy of the *SMN1* gene, named *SMN2*, is present [9], producing about 10% of a functional full-length protein due to alternative splicing caused by a single silent mutation [9]. In general, SMA can manifest across a spectrum of conditions, ranging from severe forms with perinatal hypotonia and respiratory distress as major aspects (the so-called SMA type 1) to young-adult onset, slowly progressive proximal weakness (SMA type 3) [8]. The number of copies of the *SMN2* gene has been identified as a major determinant of the phenotype’s severity in SMA patients, as higher copy numbers (CNs) tend to correlate with milder and later disease expression [10]. However, other possible modifying factors are being investigated, even as growing evidence from registries and natural history studies demonstrate different phenotypes in carriers of similar genotypes for both the *SMN1* and CN *SMN2* mutation [11].

In addition, in the last 7 years, SMA has witnessed the development and availability of disease-modifying treatments: Nusinersen, an antisense oligonucleotide, and Risdiplam, a small molecule, both targeting the splicing of *SMN2*; and gene therapy [12]. Nusinersen has proved to be beneficial to the motor and respiratory function in all SMA cohorts, with better results with earlier initiation of treatment and, for adult patients, in milder cases, with improvement in motor scales more frequently observed in ambulatory subjects compared to non-ambulatory subjects [13,14,15]. Clinical benefit is in fact more evident in the pediatric population—or even pre-symptomatic—as highlighted by the clinical trials [16,17]. Similar results are achieved by Risdiplam with regard to age and motor status at baseline [18] For Onasemnogene Abeparvovec, which is approved only for pediatric patients with differences among countries, clinical trials and real-world experience have demonstrated a marked improvement in event-free survival rates and motor scales, although with a certain percentage of adverse events [19].

In general, older SMA 2 and 3 patients which have reached adult age and have been treated in the last few years often experience a slowing of the progression of disease or stabilization [13]. Moreover, even when improvement is paramount, symptoms of muscular weakness still manifest throughout a longer lifespan, giving rise to new disease phenotypes that clinicians need to learn how to assess and manage [20]. Finally, the availability of therapeutic options has led to the implementation of newborn-screening (NBS) programs in several countries, although with regional differences [15], posing the dilemma of proper prescription and timing of treatment.

In this complex scenario, pharmaceutical research also focuses on complementary factors other than *SMN1* and *SMN2*, which may be involved in the disease pathogenesis, to possibly integrate the available treatments and ameliorate some aspects such as fatigue and motor endurance. In fact, fatigue is a prevalent symptom in SMA [21], more invasively impacting patients with moderate forms of disease such as SMA III patients with a certain degree of residual motor function [22]. The genesis of fatigue in SMA can be referred to various factors, including muscular atrophy and deconditioning, respiratory involvement, sleep breathing disorders, musculoskeletal deformities, psychological issues such as anxiety and depression, and pain [21]. At a cellular level, several mechanisms such as mitochondrial dysfunction [23] have been implied in generating fatigue in this disease, acting both at the axonal terminal and the muscle’s contractile machinery. 

Moreover, evidence from post-mortem studies and animal models have highlighted the primary involvement of the neuromuscular junction (NMJ) in SMA [24]. The neuromuscular junction (NMJ) is a specialized synapse linking the terminal of the motor nerve’s axon to the muscle fibers and is made up of three major components: the end of the motor nerve or pre-synaptic terminal, the muscle fiber’s membrane (or post-synaptic membrane) and the space between these two structures, the synaptic cleft. Around and within this complex, neurotransmitters (mainly acetylcholine, Ach) move based on ion currents which determine the fusion of the Ach-containing vesicles to the pre-synaptic membrane. Then, once in the synaptic cleft, nicotinic acetylcholine receptors (nAChRs) on the post-synaptic membrane bind the released Ach, which results in a conformational change in the receptor and then in a sodium flow toward the inner of the muscle membrane and potassium out-flow. This process produces an endplate potential, triggering a cascade of other ion transmissions and, in the end, the contraction of the muscle fiber. Dysfunction of the NMJ results in weakness and failure to sustain prolonged endurance [25]. As fatigue is the major manifestation of diseases of the NMJ such as Myasthenia Gravis (MG) and Congenital Myasthenia (CM), the prevalence of fatigue in SMA patients is not only not surprising but also warrants the need to determine the degree of involvement of the NMJ and its response to the available treatments; moreover, in this sense, the involvement of the NMJ in determining disease symptoms offers a possible parallel therapeutic target with complementary approaches that could take advantage of already available treatments that have consistently shown safety and efficacy in large cohorts of patients. 

This narrative review aims at providing an overview of the current knowledge about the pathogenesis and significance of NMJ dysfunction in SMA, including circulating biomarkers to assess its integrity, outcome measures and targeted therapeutic approaches in development.

## 2. Methods

The search was conducted on PubMed, Cochrane and ClinicalTrials.gov databases, using keywords for SMA, neuromuscular junction, biomarkers, fatigue, and clinical trials. Search terms were “Spinal Muscular Atrophy, neuromuscular junction, Myasthenia Gravis, Congenital Myasthenia, Clinical trials for SMA in ClinicalTrials.Gov, Nusinersen, Risiplam, Onasemnogene Abeparvovec, Myasthenia gravis treatment, Congenital Myasthenia treatment, fatigue outcome measures, neuromuscular junction damage biomarkers”. Review articles, in vitro studies, animal model studies, human pathology studies, clinical trials and original research papers from real-world evidence of treatments were included. Due to the narrative nature of our review, no data analysis was performed. No chronological range was set to include all the available evidence in literature, although papers from the last 5 years were prioritized when available. Only manuscripts in English were included. 

## 3. The Role of the Neuromuscular Junction in SMA

### 3.1. Pre-Clinical Studies

The NMJ is a complex structure in which neurotransmitters, ions and receptors orchestrate the transmission of the cholinergic input to the muscle and its translation in the event of muscle contraction; its formation and maintenance depend on several interplaying factors [26]. The pathogenesis of NMJ abnormalities in SMA is still debated. Since the generation of the first mouse models of SMA neuropathology in the early 2000s, a complex degenerative involvement of the NMJ and peripheral axons of motor nerves, alongside lower motor neurons, has been described [27,28], in particular as the loss of pre-synaptic nerve terminals with the accumulation of neurofilaments (NFs) and reduction in post-synaptic acetylcholine receptors (AchRs), with significant differences among different muscular groups. These findings suggested that NMJ damage could be a primary event in the disease in parallel with motor neurons degeneration. Notably, Ling et al. [29] demonstrated severe denervation in previously innervated axial muscles compared to other muscle groups without significant differences in NF levels at the NMJ between them and referred their findings to defective synaptic maintenance; interestingly, the phenotype was reversed by the administration of trichostatin A (TSA) treatment. In 2012, Osborne et al. [30] developed a series of mutant mice encompassing the whole phenotypic spectrum due to genetic engineering with variable *SMN2* CN, from none to eight, and described normal formation of the NMJ alongside later abnormalities, such as clustered AchRs, thinning of nerve terminals, nerve sprouting and, at an electrophysiological level, reduced synaptic transmission efficacy. Notably, they did not observe significant differences compared to wild type in axon loss from the ventral roots, supporting the pathological hypothesis of a primary “dying back axonopathy” in SMA. Several other animal models have tested this hypothesis, as well as a later in vitro model developed from SMA patient-derived iPSCs [31], which confirmed impaired AChR distribution on myotubes and the poor arborization of distal axons in nerve terminals in the absence of motor neuron loss. In the pre-treatment era, an elegant study by Kariya et al. [32] gathered evidence through a mouse model of the need for SMN presence in the first days of life, corresponding to the period of NMJ formation, while its depletion in later stages, after the NMJ completes development, is less detrimental, suggesting that SMN-enhancing agents would be most effective in the earliest stages of disease before the maturation of NMJ and could be conversely tapered in later stages. Later, another mouse model was used to assess the role of SMN depletion in the abnormalities observed in glial cells, which have a role in myelination and appear to be altered in SMA [33]. In this study, the restoration of SMN provided both improvements in the myelination levels and NMJ pathology in terms of an augmented number of motor terminals reaching the plate in the absence of significant differences on the skeletal muscle end, which in turn, similarly to motor neurons, did not express significant improvement. Notably, the authors also demonstrated that the overexpression of SMN was not detrimental to the tissues in the study. In 2017, Kim et al. [34] highlighted an effect of SMN depletion on levels of Agrin, which is a crucial regulator of NMJ development. The repletion of Agrin was associated, in this model, with an improvement in muscle fiber size, post-synaptic NMJ area, reduction in the accumulation of neurofilaments and the innervation of muscle fibers, at an independent rate from SMN levels. Similarly, Tisdale et al. [35] described the role of U7 small nuclear ribonucleoprotein (snRNP) in NMJ integrity. These factors seem to be dysregulated in SMA mice and are associated with denervation, reduced synaptic transmission and, again, decreased Agrin levels. Another potential modifier in disease severity was identified by Schellino et al. [36] in the c-Jun NH2-Terminal Kinase (JNK) pathway, which was found to be involved in neurodegeneration processes in SMA by Genabai et al. [37]. Inhibition of this pathway was effective in rescuing NMJ size and function in this study on mice. 

Evidence coming from functional studies suggest also other mechanisms such as mitochondrial dysfunction, which may be an SMN-depletion downstream effect, impacting not only on muscle but also on the axon terminal and the NMJ function [38]. The loss of the SMN protein has a detrimental effect on critical mechanisms of mitochondrial function, which is demonstrated by altered oxidative stress levels and ATP production [39]. Abnormalities of the morphology and function of mitochondria, as swelling and reduction in membrane christae, have been observed. Overall, subsequently to the broad effect of SMN dysregulation, mitochondria fission and fusion processes, Ca^2+^ signaling and protein production have been described as altered [39]. 

**Figure 2:**
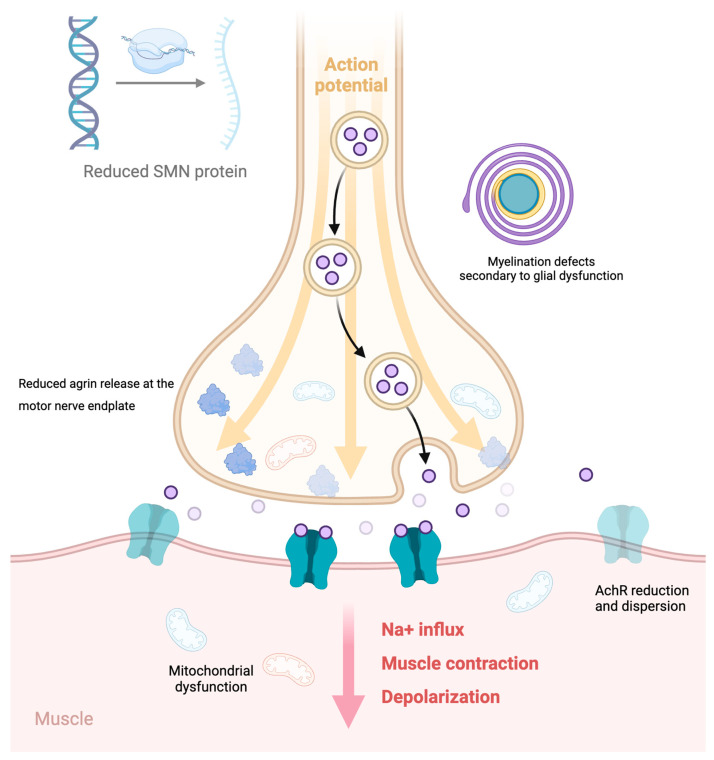
The main pathologic mechanisms involved in NMJ dysfunction (defective myelination, altered distribution of AchRs and Ach release, reduced agrin release at the motor nerve endplate (lighter shaded receptors and molecules), mitochondrial altered biogenesis and function at the motor nerve endplate and in the muscle fiber—indicated by blue mitochondria) upon reduced SMN levels are reported. Figure created with BioRender (https://app.biorender.com/ accessed on 28 June 2024).

The proposed pathological mechanisms are summarized in Figure 2.

### 3.2. Clinical Experience 

A first clinical observation of the involvement of NMJ in SMA was carried out on 35 SMA patients versus 20 healthy controls and 5 controls with motor neuron disease, which were tested with repetitive nerve stimulation. The study highlighted a pathologic decremental response in nearly half of the SMA patients and not in the control groups although without correlation with disease type and duration [40]. Conversely, Bartels et al. [41] did not report significant NMJ disfunction in their study that evaluated a cohort of 61 pediatric and adult SMA patients for fatigability through the Endurance Shuttle Test Combined Score (ESTCS), strength (MRC sum score), motor function (with the Hammersmith Functional Motor Scale Expanded—HFMSE—and Motor Function Measure—MFM) and NMJ function with repetitive nerve stimulation of the accessory and ulnar nerves along with patient-reported questionnaires. The authors impute this finding to a high number of missing values in the clinical and electrophysiological evaluations. Still, fatigue and reduced motor endurance, which are generally present in diseases characterized by prominent NMJ dysfunction, represent a major complaint for SMA patients, although controlled trials specifically assessing the dimension of fatigue and its clinical and biochemical relationship with SMA treatments are lacking. 

In 2019, Montes et al. reported increased motor endurance in SMA patients treated with Nusinersen tested with the 6MWT [42], although with differences among different age stages, which was hypothetically because of the effect of Nusinersen on neuromuscular junction dysfunction. Also, Kizina et al. evaluated fatigue in SMA subjects undergoing treatment with Nusinersen: in their study, fatigue appeared as a significant burden showing some degree of response to treatment with Nusinersen [43]. Another study, on the other hand, did not highlight a direct relationship between perceived fatigue and motor endurance [44]. Recently, Ricci et al. have developed a clinical scale aiming at assessing motor endurance at upper and lower limbs through combining several repetitive tasks which were already present in other motor scales [45]. 

Thus, larger cohort studies correlating circulating biomarkers, neurophysiological findings, motor function and patient-reported outcome measures (PROMs) are strongly needed to assess the burden of fatigue and the clinical relevance of NMJ dysfunction.

To date, the main considered biomarkers for SMA include SMN transcript and protein levels [29]. Considering MG, in which the NMJ damage is the predominant pathophysiological event, circulating biomarkers paralleling disease severity and response to treatment are lacking. Serum acetylcholine receptor α1 subunit protein [46] was analyzed in a cohort of anti-AChR antibody positive MG patients in which higher levels were detected, which was possibly related to the NMJ damage; in this condition, the increase could be due to the endplate damage determined by the autoantibodies [47]. Taken as an indicator of NMJ disruption, it could be interesting to study this biomarker also in SMA, correlating it to endurance tests performance and electrophysiology. 

## 4. Therapeutical Approaches Targeting the NMJ in SMA

To date, the main therapeutical options for SMA are directed at restoring SMN levels [48] along with other complementary approaches [49,50]. Among the already existing and used treatments in SMA, ß2-adrenoceptor agonists like salbutamol, valproic acid, pyridostigmine, and other medications used in CM can be listed. 

At present times, the treatments’ pipeline for SMA includes several strategies other than other gene therapy solutions and SMN-replacing molecules, such as enhancing muscle fibers growth through the inhibition of myostatin [51] and targeting ion channels at the NMJ to enhance its function [52]. 

Salbutamol, a short-acting β2 adrenergic receptor agonist classically used to treat asthma and chronic obstructive pulmonary disease, has been offered to patients with SMA and other neuromuscular diseases for many years with empirical knowledge of its beneficial effect well before controlled trials were performed. The efficacy of salbutamol on enhancing NMJ function in both improving motor endurance and reducing the decrement in CMAP amplitude after repeated stimulation may be related to the observed increase in *SMN2* transcript length and level with a direct relationship with *SMN2* CN [53,54,55,56]. 

Similarly, valproic acid, acting on multiple ion channels (calcium and sodium), modulating GABA neurotransmission, and histones deacetylases (HDACs) has been employed in SMA in the last few years without strong evidence of efficacy in ameliorating motor function [57] or to increase SMN protein levels with contrasting evidence for this parameter and its relationship with a better function of NMJ [58,59,60,61]. Lately, the action of VPA on HDACs has been re-evaluated considering its possible synergistic action with Nusinersen [45]. 

Another compound, flunarizine, was tested in a pre-clinical setting and improved NMJ function by increasing Agrin and Agrin receptors levels [62].

Conversely, pyridostigmine, the commonly used treatment in Myasthenia Gravis (MG) acting as a neuromuscular excitation enhancer [63], has been tested in SMA with positive results in a placebo-controlled trial on patients with SMA types 2–4, demonstrating an improvement of motor endurance despite stable motor function and strength [64]. These results were further confirmed in a subsequent electrophysiology study [65] involving 31 SMA type 2 and 3 patients that underwent surface electromyography (sEMG) while performing endurance shuttle tests (ESTs) and maximal voluntary contraction measurements; patients taking pyridostigmine showed significantly lower decreases in sEMG frequency and smaller increases in sEMG amplitude, indicating improved low-threshold motor unit function without the need to activate further motor units or increase firing rates.

More recently, pre-clinical evidence of possible efficacy on an in vitro model was gathered [66] about a possible beneficial role of nifedipine, a dihydropyridine (DHP) antagonist of L-type Ca^2+^ channels, which has been demonstrated to increase evoked neurotransmission in animal models [67,68] and to increase spontaneous release in motor nerve terminals through the enhancement of intracellular Ca^2+^ stores transfer [49]. In their experiment, Tejero et al. demonstrate in a culture of spinal cord motor neurons from control and SMN-deficient mice that the administration of nifedipine increased NMJ function and evoked a spontaneous release at low-frequency stimulation in both groups, while high-frequency stimulation only increased the NMJ function in controls. Further evidence is necessary to successfully apply this already available, safe compound. 

Another product borrowed by MG and CM is 3,4-diaminopyridine phosphate (3,4-DAP) [69,70], which is a voltage-dependent K^+^ channel blocker that enhances neuromuscular transmission by an elongation of pre-synaptic terminals’ depolarization, thus increasing acetylcholine release. In SMA, its potential application was recently tested by Bonanno et al. [71] in a cohort of 13 SMN-enhancing treatments’ naïve patients, in which the Hammersmith Motor Functional Scale Expanded (HMFSE), 6MWT, the time to rise from floor, the time to rise from chair, the time to climb four standardized stairs, and the time to walk 10 min were tested. Treatment with 3,4-DAP determined higher scores at HMFSE but no significant changes in the other measures. Notably, as the authors point out, patients reported that fatigue—through the Individualized Neuromuscular Quality of Life questionnaire (INQoL) scale—improved with treatment. 

Other molecules are applied to CMs with different rationales [72]. For example, fluoxetine, a selective serotonin reuptake inhibitor antidepressant, is used in patients with slow-channel syndromes [73] due to its role as a long-lived open-channel blocker of the AChR ion channel, resulting in prolonged synaptic transmission due to shortened synaptic currents and reduced desensitization of the receptor; quinidine as well, an antimalarial and a class Ia antiarrhythmic agent, exerts similar effects at the neuromuscular junction [74]. To date, to our knowledge, no trials or anecdotal experience for fluoxetine or quinidine application in SMA have been reported in the scientific literature nor in the ClinicalTrials.gov database. 

All the mentioned compounds have been used for several years in chronic neurological disorders and other conditions, and their use is backed by theoretical and clinical practice knowledge about dose titration, adverse events, and safety concerns. For example, valproic acid has recently been warranted a safety warning not only in women of childbearing age but also in males due to increased risk of autism in offspring [75], so it may be more suitable for older patients or for subjects without reproductive desire; medications such as flunarizine, salbutamol, pyridostigmine and 3,4-DAP can be contraindicated in cases of heart conditions and arrythmias in particular, which may hamper their use in adult age; fluoxetine is not recommended for young patients, as it may trigger mood disorders and suicidal ideation [76] and is contraindicated during pregnancy [77], which may be increasingly considered by female SMA patients. Given the broad range of disease severity in SMA and the changing phenotypes from using disease-modifying drugs, these complementary approaches should be tailored to the patient’s age, comorbidities, and wills. In general, in most countries, the cost-effectiveness of these treatments is moderate to high, so their use in clinical practice as add-on options could be feasible. 

A phase II clinical trial [53] is recruiting ambulant, SMA III patients to test NMD-670, a molecule acting on a skeletal muscle specific chloride (Cl^−^) ion channel involved in neuromuscular transmission and muscle fiber excitability. This molecule, after a successful phase I trial, has been tested on patients with MG, reporting a favorable safety profile and clinically relevant improvements in the Quantitative Myasthenia Gravis (QMG) total score [78]. The clinical trial for SMA patients will assess clinical parameters such as the 6MWT and the Motor Function Measure 32 scale (MFM32) along with electrophysiological parameters through single-fiber EMG, and PROMs. 

Concerning the effect of the already approved treatments on NMJ function based on their primary effect of increasing SMN protein levels, Nusinersen has shown, in SMA mice, to rehabilitate NMJ morphology and function, fostering synaptic maturation, improving neurotransmission, and boosting muscle innervation [33]. According to these findings, Nusinersen could potentially improve neuromuscular transmission, muscle strength, and functional outcomes by exerting beneficial effects on the NMJ in SMA patients. Similarly, in pre-clinical studies, it has been demonstrated that Risdiplam treatment enhances the integrity and function of the NMJ in SMA animal models, which leads to an increase in synaptic area, enhanced neuromuscular transmission, and improved muscle innervation [79]. Nevertheless, patients treated with “SMN-replacing” molecules also show some degree of disease progression, indicating that SMN depletion may not be the only factor responsible for all the downstream effects, including NMJ dysfunction [80].

Overall, clinical trials for the complementary approaches have so far involved small cohorts of subjects, while the NMJ function has not been considered as a main outcome in trials on the approved treatments; this adds to the lack of homogeneity of assessment (endurance motor tests, functional tests, PROMs, electrophysiology, biomarkers, and others). Given the evidence of the significant involvement of NMJ in determining SMA symptoms and the availability of numerous and diverse therapeutic options, which have already been proved safe in other diseases, it would be advisable to design more ample clinical trials for the eventual application of these drugs. The discussed treatments are reported in Table 1. 

## 5. Outcome Measures for NMJ Involvement

As expected from the pathophysiological mechanism, the main clinical manifestations of NMJ dysfunction are perceived fatigue and reduced endurance to repetitive motor tasks [44]. The two aspects my need to be distinguished and separately assessed, as they refer to various mechanisms involving the psychic sphere, central nervous system, peripheral nervous system, and metabolic processes. In general, perceived fatigue is assessed through PROMs in the form of questionnaires, while motor endurance is often assessed through standardized motor tests requiring the subjects to endure prolonged exercise or repeated tasks. 

### 5.1. Patient-Reported Outcome Measures for Fatigue in SMA and NMJ Disorders

The most common PROMs also used in SMA include the Fatigue Severity Scale (FSS), a self-report questionnaire investigating the impact of fatigue in daily life [81]; the Visual Analog Scale (VAS), a simple visual tool in which patients rate their level of fatigue from 0 to 10 [82]; and the Multidimensional Fatigue Inventory (MFI), assessing the fatigue dimension in multiple domains as physical and mental fatigue [83]. Moreover, quality of life questionnaires such as the InQoL [84] often include sub-groups of questions assessing fatigue. In the case of MG, specific clinical scales as the Quantitative Myasthenia Gravis (QMG) scale, the MG Activity of Daily Living (MG-ADL) scale and the MG Quality of Life 15-items (QOL15) are used in clinical practice and trials, also evaluating speech, swallowing and respiratory function, which could be possibly applied also to SMA [85].

### 5.2. Timed Motor Tests as Outcome Measures for Fatiguability

Concerning motor endurance, one of the tests most often applied to clinical practice and trials is the six-minute walk test (6MWT): a timed task in which the subject is asked to walk along a 25 mt long path for 6 min. The evaluator assesses the global length the patient covered and the time at each “turn” over the 25 mt path as well as falls and stops. Variations in speed of the subject over the time of the test can be evaluated and provide a measure of fatigability [86]. This test has been used in several neuromuscular diseases, as well as clinical trials’ endpoint, including SMA [72]. The Timed Up and Go (TUG), which measures the time needed to stand from a chair, walk a 3 m distance, walk back, sit and repeat the task for a variable number of times, is another useful indicator of fatigability, especially in SMA and conditions characterized by major involvement of the proximal muscles of lower limbs [87]. These timed motor tests have the obvious limitation of being suitable only for ambulatory patients, while for the upper limbs, the Nine-Hole Peg Test (9-HPT), evaluating digital dexterity as the patient repeatedly picks up, inserts, and removes nine pegs from a peg board with holes in a timed framework (so that both the total number of “laps” and single durations can be assessed) can be used also in “standers” or “sitters” categories of SMA patients [88]. More severe subjects with only little motor function preserved and joint contractures lack adequate motor outcome measures, and they may benefit more from electrophysiology studies, digital outcome measures and circulating biomarkers instead. 

### 5.3. Digital Outcome Measures for Fatiguability

The availability of novel outcome measures provided by digital tools and remote monitoring could represent a useful data source for prolonged observation and for precisely objectifying fatigue and its clinical correlates. For instance, surface electromyography, gait analysis, and video-based motion analysis tools could provide a wide amount of information both in clinical practice and trials. These kinds of data, that can be acquired through cameras and wearable devices, could in fact permit the observation of subjects in real life and for longer periods out of the experimental setting. One pilot study of 18 SMA 3 patients and 19 healthy age-matched controls demonstrated the feasibility of the application of the Microsoft Kinect sensor that, through a specifically designed game, captured upper limb movement, allowing the detailed analysis of joint motion limitations in a cohort of SMA type 3 patients [89]. The authors did not find a distinction among patients and control when considering joint angles, which is not surprising considering the cohort of SMA type 3 patients, while SMA patients reported better scores in hand velocity tasks. These results need to be confirmed in larger cohorts and with follow-up evaluations. Another study utilized the ActiMyo device to measure physical activity in non-ambulant SMA patients, recording upper limb movements in daily life, revealing decreased wrist vertical acceleration in sitting SMA type 2 patients compared to non-sitters [90]. At our center, we are currently testing a system of wearable devices for both the upper and lower limbs (AUTOMA, [91]), including surface EMG, accelerometers and goniometers on subjects performing an endurance-targeted motor scale, which is intended for use on SMA type 3 patients. Information coming from wearable devices and digital tools in general has the potential to assess fatigue and loss of endurance during daily activities in SMA patients. 

### 5.4. Electrophysiology Parameters 

Also, electrophysiologic parameters have been used in the past few years to assess the NMJ in SMA, both in trials and pathophysiology studies, and are being applied to new clinical trials [53]. A major indicator of NMJ dysfunction is single-fiber electromyography (sfEMG), which provides a quantification of neuromuscular transmission through the jitter analysis [92]. Noto et al. [93] analyzed fatigue and stimulated-sfEMG in a cohort of spinal bulbar muscular atrophy (SBMA) and SMA patients and controls affected by other neurological disorders associated with fatigue. The authors concluded that an activity-dependent conduction block was present in SBMA/SMA patients, although they could not identify the site of blocking (the axon, NMJ or muscle), and their methodology was limited by lack of a proof by the administration of pyridostigmine. As reported above, sEMG was also used to assess motor unit function [66], as well as repetitive nerve stimulation by Bartels et al. [41], without univocal conclusions. Overall, the lack of standardized assessment in homogenous cohorts hampers the possibility of gaining stronger insights on the mechanisms of damage and response to treatment of the NMJ. 

Considering the absence of multiple established circulating biomarkers and the available evidence from the literature, it would be advisable to combine clinical outcome measures for motor endurance, electrophysiology and the parameters that are usually considered or have been implied in experimental settings, such as *SMN2* transcript or the acetylcholine receptor α1 subunit protein, in order to identify reliable markers of disease progression and response to treatment. 

The considered outcome measures are summarized in Table 2.

## 6. Conclusions

While stunning results have been obtained in clinical improvement for SMA since the availability of SMN-targeted therapies, as patients experience increased survival, reduced need for ventilatory assistance and age-adequate motor milestones [16,17,18], NBS and prolonged lifespan pose new challenges in treatment decision making and the management of novel phenotypes and symptomatology involving, among others, the need to manage musculoskeletal conditions as scoliosis—which can be linked to respiratory issues, cognitive delay especially in type I forms, and chronicity-associated complaints, such as pain and fatigue [94,95,96]. In this sense, the long-standing evidence supporting the involvement of the NMJ in the SMA clinical picture and the availability of numerous and safe therapeutical approaches that could be applied from other conditions such as MG and CM offers the opportunity to address patients’ needs and improve quality of life, impacting important symptoms such as fatigue and motor endurance. Moreover, to our knowledge, no drug–drug interactions should be expected with the available disease-modifying therapies. In addition, clinical trials for therapeutical options targeting NMJ are ongoing. To reinforce the indication to apply already available and known therapies, and to develop novel approaches, the extent and significance of the NMJ involvement in the various subtypes of patients (i.e., according to genotype, clinical manifestation, etc.) should be further and systematically investigated though multiparametric standardized studies, including electrophysiology, motor tests and PROMs. In this framework, observational studies and clinical trials should be performed and correctly designed to include wide cohorts of homogenously selected patients at least for age, genetics, phenotype, and concomitant medications. Moreover, circulating biomarkers for NMJ damage, able to ascertain the efficacy of treatments on this component, should be further explored as potential markers of treatment efficacy and disease progression.

## Figures and Tables

**Table 1:** Different therapeutical approaches targeting the NMJ.

Drug	Mechanism of Action	Applied to SMA
Salbutamol	Increased *SMN2* transcript	Yes
Valproic acid	HDACs modulation	Yes (discordant data)
Pyridostigmine	Neuromuscular excitation enhancer	Yes
Flunarizine	Agrin and agrin receptors enhancer	Yes
Nifedipine	Neuromuscular excitation enhancer	Yes
3,4-DAP	Neuromuscular excitation enhancer	Yes
Fluoxetine	Prolonged synaptic transmission	No
Quinidine	Prolonged synaptic transmission	No
NMD670	Chloride (Cl^−^) ion channel inhibitor	Yes (clinical trial ongoing)

**Table 2:** Outcome measures (PROMs, motor tests, digital tools, and electrophysiology) useful for the assessment of the NMJ.

Outcome Measure	Type	Applied to SMA
FSS	PROM	Yes
VAS	PROM	Yes
MFI	PROM	Yes
InQoL	PROM	Yes
6MWT	Timed Motor Test	Yes
TUG	Timed Motor Test	Yes
9-HPT	Timed Motor Test	Yes
MG specific PROMs	PROM	No
ActiMyo, Kinect, AUTOMA	Wearable devices/digital assessment tools	Yes
sEMG, sfEMG	Electrophysiological tests	Yes

## Data Availability

No new data was generated within this manuscript.
